# Flexibility in rigid systems: a meta-synthesis of best practices for integrated care

**DOI:** 10.1186/s12875-025-03062-y

**Published:** 2025-11-11

**Authors:** Sofia Backåberg, Mirjam Ekstedt, Elin-Sofie Forsgärde, Heidi Hagerman, Kristina Tryselius

**Affiliations:** ᵃDepartment of Health and Caring Sciences, Linnaeus University, Universitetsplatsen 1, 392 31 Kalmar and Växjö, Sweden

**Keywords:** Care transitions, Integrated care, Collaborative reflexive deliberate approach, Flexible caring, Learning organizations, Patient-centeredness, Patient safety

## Abstract

**Introduction:**

Integrated care has the potential to mitigate patient safety risks by enhancing collaboration and maintaining a patient-centred approach. However, best practices for successful implementation are lacking. This study aims to identify and describe key components of best practices for integrating health and social care to increase understanding of successful implementation.

**Methods:**

A Collaborative Reflexive Deliberative Approach was used. The data comprised twenty-one published articles and five unpublished manuscripts from 2015 to 2023, along with the experiences of ten clinicians and researchers in integrated care, and the research team itself.

**Results:**

Components identified as best practices for integrated care, each describing different aspects shaped by and for the patient, were: holistic co-creation in an ethical stance, trust through physical and relational proximity, flexible caring, learning and adaptable organizations and flexible information and communication.

**Discussion/conclusion:**

The study emphasizes the importance of building trust through proximity and adaptable organizational learning, and the need for a holistic perspective, acknowledging both the limitations and potentials of health and social care integration. Embracing innovative thinking and recognizing that not everyone needs all services at all times can foster flexible, person-centred integrated care. Addressing these complexities is essential for successful integration efforts.

**Supplementary Information:**

The online version contains supplementary material available at 10.1186/s12875-025-03062-y.

## Introduction

Globally, the health and social care systems are structured into specialized areas of responsibility. Such specialization enables professionals to become experts in specific domains. However, it creates challenges in the form of fragmentation, which may potentially limit a comprehensive understanding of patients’ situations and health [1–3]. Patients with complex care needs are particularly vulnerable, as their care requirements often involve multiple professionals from different organizations. Fragmentation creates risks for patients, such as gaps in information and care during transitions from one setting to another [4].

Integrated care addresses fragmentation by connecting and coordinating health and social care organizations [5]. Integrated care is a concept with multiple definitions and contents [6]. It is influenced by the different perspectives and expectations of users in the health system, making it difficult to define uniformly [7]. However, enhancing collaboration within and between different health and social care organizations, along with adopting a more integrated, people-centred approach to care delivery, has the potential to generate significant benefits for the patient [8]. Effective collaboration and communication among professionals are described as crucial for a comprehensive understanding of patient’s health and social care needs [2]. At times, health and social care successfully integrate care through shared intentions and agreements. However, such collaboration across organizations is underdeveloped in many countries [4, 9, 10]. The patient perspective is widely recognized as the foundational principle in integrated care, offering an alternative to organization-driven models [10]. From the patient perspective, integrated care is described as personalized and coordinated [11], fulfilling care needs within a reasonable time frame [12] and increasing satisfaction with and improving experiences of quality of care [13]. However, a lack of comprehensive synthesis describing the best practices of integrated care remains.

There are several reasons for health and social care organizations to strive towards integrating care services, such as enhancing patient care, improving health population outcomes, and achieving organizational efficiency goals [11]. A meta-analysis identified an overall association between integrated care, lower costs, and improved outcomes compared with ordinary care [14]. However, research on the cost-effectiveness and outcome of integrated care shows differing results [13, 14] and evidence on the effectiveness of integrated care remains limited [4]. Nevertheless, as described above, integrated care holds promise for enhancing patient experiences, fostering collaboration, and mitigating fragmented care by addressing the needs that emerge during transitions of care across organizations [1, 4]. Continuity of care is described as a critical component to enable integrated care and appears to be advantageous for achieving a comprehensive understanding of patient health and social care needs [15–19].

Current literature shows that identifying fragmentation in the care for patients within and across organizations is crucial to increasing the quality and safety of care [4, 9, 10]. There are several national and international examples of efforts to address these challenges by striving to implement integrated care [10, 20, 21]. These efforts provide valuable insights into the potential for systemic change, yet significant barriers to the successful implementation of integrated care remain, resulting in few successful examples [20–22].

This collaborative study addresses these challenges by synthesizing existing knowledge with the aim to identify and describe key components of best practices for integrating health and social care to increase understanding of successful implementation. Building on the World Health Organization’s [23] definition, this study expands the concept of ‘best practice’ to incorporate patient involvement. *This means a practice that becomes as effective as possible through the responsible use of available resources, is shaped by the patient’s health status, needs, and values, and involves techniques or methodologies that have proven to reliably lead to the desired results through experience and research.*

## Methods

### Design

This study is part of the Resilient Healthcare and Patient Activation (ReAction) research program at Linnaeus University, Sweden. The program focuses on exploring cross-organizational collaboration and care coordination to co-create integrated care for people with complex care needs. In this study, complex care needs refer to individuals who require care from various health and social care providers, primarily delivered at home. The ReAction group has conducted extended research, revealing the need to synthesize data from individual studies, which often involve small sample sizes. This approach allows for the examination of problem areas from different angles and enhances the robustness of the findings, which helps build a strong evidence base for future research [24]. In this study, synthesizing included mapping and visualizing interdependencies, gaps, adaptations, and collaboration in integrated care across health and social care settings. The results from this study have the potential to guide future interventions involving the design, implementation, and evaluation of best practices for integrated, person-centred, and timely care for those who need it most.

The study followed a Collaborative Reflexive Deliberative Approach (CRDA), as developed by Crabtree et al. [25]. The goal of a CRDA is to synthesize and develop knowledge that goes beyond what has previously been published in a field, providing “a richer description of what lies between the lines of published studies” [25], p. 273). Following the method, the current study was conducted in an iterative process with repeated collaborative face-to-face, and online meetings. The method was slightly modified since no unanalysed data relevant to the study existed, hence not included.

### Context and sample selection

Sweden's healthcare system, in which these studies are conducted, is decentralized, with regions and municipalities managing different parts. Regions are responsible for primary, secondary, and specialist care, including healthcare centres and rehabilitation services. Municipalities handle home healthcare and social care, such as care for older people, nursing homes, and home care services. The bulk of health and social care services in Sweden are funded by regional and municipal taxes and delivered by public or private providers. The core principle is to ensure that services are accessible to all individuals who require care, irrespective of their financial situation or family support. Although the national government sets an overarching political direction and enacts laws and regulations, regions and municipalities operate autonomously. This self-governance allows them to tailor services to the specific needs of their local populations [26]. For individuals with multiple or complex health and social care needs who are primarily cared for at home, a well-established collaboration between various regional and municipal care providers, as well as between primary and secondary care, is essential.

In line with the CRDA, the research team members were considered both investigators and participants, jointly reflecting and synthesizing findings from published and unpublished studies, information, knowledge, and experiences [25] over a period of two years (2023–2024). The team consisted of five researchers (SB, ME, ESF, HH, KT) from the ReAction group. The team members had diverse research backgrounds and experiences, and various clinical backgrounds, including roles as registered nurses or physiotherapists in primary healthcare or inpatient care across different parts of Sweden. The invited collaborative researchers and clinicians were selected based on their diverse experiences and perspectives of integrated care across care settings in national and international research. In total, ten collaborative researchers from the Nordic countries, the UK, and the Netherlands were invited. Five consented to participate, but three had to withdraw, resulting in two participants. Twelve patients, family carers, and clinicians were invited of which eight participated. The participants included one coordinator for family carer support, one social worker, one general practitioner, one physiotherapist, one assistant nurse, one care administrator, and two registered nurses. However, no patients or family carers consented to participate.

### Research phases in CRDA, data collection, and analysis

The CRDA includes four phases: A) refining peer publications (select, focus, extract), B) organizing and adding broader study materials (classifying, re-extracting, analysing), C) interpreting and adding external collaborative knowledge (analysing, absorbing, re-interpreting), and D) integrating and adding experiential reflections (re-interpreting, reflecting, integrating) [25].

#### In phase A

The dataset of peer publications was refined. The dataset encompasses data from diverse empirical care settings and levels (micro, meso, macro). The selection process involved screening a total of 154 research endeavours (including research projects, post-doctoral studies, and PhD dissertations) conducted between 2014 and 2023, based on the broad theme ‘qualitative studies on seamless, coordinated care or continuity of care for patients with complex needs. Projects with other scopes, and quantitative studies were excluded. This screening process culminated in a sample of 26 articles. Selected articles were discussed at a two-day meeting, followed by sorting, concept mapping, and refining the research aim to ‘synthesize findings from various settings to identify best practices for patient-centred integrated healthcare’. Subsequently, 21 relevant peer-reviewed articles from the ReAction group (2015–2023) were selected. In a second two-day meeting, key concepts and themes were extracted and organized using coloured cards.

#### In phase B

Broader materials in the form of five unpublished manuscripts, soon to be submitted, were added, expanding the dataset to 26 articles and manuscripts in total (Appendix 1). All data were then classified corresponding to the re-focused aim and extracted into both existing and new cards. The analysis and reflections within the research team involved further concept mapping and recognizing similarities, differences and relationships among the extracted data.

#### In phase C

Knowledge from collaborative researchers and clinicians was added to data. Two face-to-face workshops with clinicians and one online workshop with researchers were held. At the workshops, the participants were asked to individually write down their experiences related to the re-focused aim and then discuss them together. The knowledge gained was documented on Post-it notes and reflective field notes by the participating research team members. The research team performed in-depth analyses of the outcome directly after each workshop. After all workshops had been conducted, the research team gathered face-to-face to integrate the added knowledge, re-interpret all the relevant data, and review the refined outcomes of the analyses. Summaries of findings were then created.

#### In phase D

The research team met in a two-day face-to-face meeting, in which each research members’ individual perspectives, deriving from knowledge and lived experience, and summaries and reflections of the findings thus far, were shared in an open discussion. At this point, the final findings were formulated into five components of best practices for integrated care. This allowed all research members to compare the insights from the current findings within a broader context before jointly formulating the final meta-synthesis [25].

## Results

The five components of best practice for integrated care; *holistic co-creation with an ethical stance*, *trust through physical and relational proximity*, *flexible caring, learning and adaptable organizations* and *flexible information and communication*, describe characteristics shaped by and for the patient.

### Holistic co-creation with an ethical stance

Best practices in integrated care are permeated by an ethical approach characterized by a holistic view of the human being, not excluding any aspect of life. It involves a bird’s-eye perspective, which means being able to see the whole system, not just one part. Creating a sense of wholeness and togetherness – that ‘we are in it together’ – is a prerequisite for shaping a care system that can enable best practices for patients and next of kin. Best practices for integrated care also include overarching patient-related goals that everyone strives for and is aware of. Here, patients and next of kin need to be seen as co-creators. When patients and next of kin choose to share their stories, best practices mean professionals are aware of their responsibility for respecting that confidence. The experiences that patients and next of kin convey about qualitative aspects of care can work as sensors of the quality of care – both when things go well and when they go wrong or not as intended. However, this requires that professionals listen to those experiences.

### Trust through physical and relational proximity

Fundamentally, best practices in integrated care involve patients and their next of kin experiencing trust in both the care systems and the professionals within them. Trust is fostered through meaningful interpersonal relationships facilitated by physical and emotional proximity. Proximity is established when people get to know each other, building a shared and reciprocal understanding. This becomes a prerequisite for building trustful relationships in both in-person and remote interactions, regardless of physical distance. A patient’s perception of proximity depends on professionals creating a sense of presence, regardless of the spatial and temporal conditions of an encounter. Professionals build trusting relationships with patients and their next of kin by showing respect, engagement, active listening and genuine concern for each patient’s needs. When patients and their next of kin feel seen and respected, they trust that the professionals will uphold their promises. A trusting relationship can positively impact a patient’s adherence to treatment, sense of security and active participation in their own care.

Accessibility to health and social care is a crucial component of the experiences of patients and next of kin – care should be available when needed and delivered in a tailored manner. Team collaboration at various levels within the care system is essential to provide the necessary care, regardless of care provider. In best practices, professionals place trust in the system and each other, confident that the next instance will smoothly take over where their own responsibility ends. Familiarity among professionals possibly bridges gaps within and between organizations, particularly during care transitions. Achieving this requires that professionals understand each other’s roles and mandates. In organizations where there is trust in managers and colleagues, professionals have the courage to take responsibility beyond their own areas and comfort zones, extending their impact beyond existing boundaries. Integrated care is fostered by an atmosphere of trust, proximity and reciprocal relationships, where organizational and spatial boundaries have little impact.

### Flexible caring

A central component of best practices for integrated care is what might be termed flexible caring. This approach and behaviour should be used from a patient’s initial contact with health and social care services and continue as long as care is required. A prerequisite for flexible caring is that professionals are aware that plans formulated for a patient’s care, such as standardized care plans, discharge plans, guidelines and routines, are theoretical and may not align with the patient’s actual reality. Personalized care plans that are created after deliberation and with built-in flexibility can play a crucial role in promoting continuity and integration throughout the care process.

However, the opposite is also evident – care plans can be inhibiting if they allow no space for flexible caring. In practical care situations, flexibility means that different professionals must be able to make independent, context-specific assessments and formulate new plans based on the current circumstances of patients and next of kin. A flexible use of standardized tools and policy documents also reflects an awareness that an individual’s life situation and care trajectory are dynamic, necessitating adaptability in documentation and assessments.

Time is a central aspect of flexible caring. Professionals should create an experience for the patient, next of kin, and themselves of having the time and space required to reflect, understand, and communicate current care – regardless of the actual time they have available. Timing is also crucial, and patients and next of kin should be asked what constitutes timely care for them.

Flexible caring requires professionals to act dynamically and adapt to the situations, environments and people they encounter. A prerequisite for handling, e.g., standardized care tools flexibly is that all forms of caring actions begin in the encounter with the patient and next of kin. In conclusion, flexible caring as a component of best practices for integrated care can be said to elevate the fundamental principles of care by referring back to the patient and the individually tailored care.

### Learning and adaptable organizations

A vital aspect of best practices, learning and adaptable health and social care organizations play a pivotal role in promoting integrated care. In a well-functioning organization, adaptability is characterized by continuous individual and group learning at all levels of the organization, with changes to the system and organization also being made based on the lessons learned. Such a learning environment requires that professionals in the organization meet and work together but are also given space to reflect on their own work and tasks. Employees, regardless of their profession or role within the organization, can then feel empowered to lead their work. They can act based on their specific expertise and areas of responsibility in an independent and adaptable way based on each unique care situation. In other words, they have a mandate for flexible caring, knowing that the organization and managers support this.

To achieve best practices, professionals also need to have the necessary skills, experience and a willingness to learn. This includes the courage to act flexibly and stretch boundaries, even if it means deviating from standardized processes through workarounds, when the circumstances require this. A fundamental prerequisite for success is that the organization ensures that there is professional competence adapted to patients and next of kin. When competence aligns with organizational roles and flexible care conditions, it fosters a sense of security among employees and promotes best practices for integrated care.

### Flexible information and communication

As a critical element of best practices for integrated care, information and communication should flow both vertically and horizontally, accompanying the patient across all levels of care. The information flow should be adapted to the individual patient needs, preferences and input from next of kin. Such an approach fosters collaboration and high-quality integrated care. It is a way to maintain patient autonomy and ensure that patients feel seen. Continuous and follow-up information and communication are therefore central components of integrated care.

Flexibility in responsiveness, which allows for determining the optimal timing for information and communication, is crucial for achieving best practices. Additionally, sensitivity to the type of information provided on each occasion is also highlighted as important. It involves discerning when patients and their next of kin are open to and have the desire and need for information. A prerequisite for professionals to do this is that they are available, present and open. For timely communication, questions about the present and future should be given priority, without ignoring the past. It is also the professional's responsibility to confirm that the patient and next of kin understand the information and review their knowledge or need for knowledge. In best practices for integrated care, timely information and communication, as well as follow-ups, are provided in both verbal and written form.

To achieve flexible information and communication, adapted for the patient and next of kin, structures and systems that enable cohesive information-sharing among professionals are necessary. Successful examples of this are when regions and municipalities use the same electronic health record system and share coordinating communication systems. Using national systems for cohesive electronic health record systems, such as the Swedish National Patient Summary, has been identified as a potential component of best practices for integrated care. For the effective functioning of information and communication processes, it is essential that professionals are familiar with the routines and organizational structures of other care providers. Spatial proximity between care providers results in shorter processing times for patients and, consequently, makes it easier to maintain a flexible flow of information and communication.

Other components of best practices for integrated care are physical and digital tools as enablers for a continuous and personalized flow of information and communication. An example is using laminated cards containing important contact details to health and social care providers, specific to each patient. Various types of digital tools, such as tablets, mobile phones and applications, are also highlighted as important. Digital health and social care is regarded a complement to face-to-face care. Personal relationships between patients, next of kin and professionals need to be established before digital communication begins. Best practices also involve assessing which interactions and conversations require physical contact and which can be carried out digitally. Flexibility and responsiveness to patients’ preferences for physical or digital meetings are essential in achieving the right balance.

## Discussion

The current meta-synthesis identifies five components making up best practices of integrated care: *holistic co-creation with an ethical stance*, *trust through physical and relational proximity*, *flexible caring*, *learning and adaptable organizations* and *flexible information and communication*. These components have been proven reliable through experience and research, and each plays a crucial role in delivering high-quality, patient-centred care. By recognizing the interconnectedness of these components, it becomes possible to see beyond individual practices and understand how they collectively contribute to a more comprehensive and effective care system. This broader perspective allows to ‘see the bigger picture’, providing a more comprehensive view of the integrated care landscape. The findings offer valuable guidance for successful implementation of best practices in the integration of health and social care, which is greatly needed.

In the following sections, we will discuss common pitfalls and challenges that impede the successful integration of health and social care services. We have identified key elements of best practices of integrated care to achieve optimal outcomes while responsibly utilizing available resources. This means that integrating all services for everyone is not feasible, in accordance with the first law of integration proposed by Leutz [27] 25 years ago, which remains relevant today. Despite resource constraints, there is an ongoing increase in demand for health and social care services. Instead of pursuing a one-size-fits-all approach, interventions should be flexible and tailored, shaped by each patient’s health status, needs, and values through holistic co-creation with an ethical stance. The question is how this balancing act can be achieved sustainably while providing high-quality care.

Professionals often grapple with ethical dilemmas that increase demands in their work practices. Delivering integrated healthcare and social services necessitates a high degree of interdependency between professionals [28], [29]. Togetherness founded in trust in each other’s competence and accountability acts as the essential glue in such endeavours. Any effort to enhance task integration – whether through shared decision-making with patients or through the integrated use of multi-professional digital care – disrupts the existing workflow, as illustrated by Leutz’s third law: “your integration equals my fragmentation” [27]. Although integrated care benefits patients, care providers perceive changes as disruptive to their established, standardized routines, fragmenting their accustomed practices. Proximity – both physical (sharing localities) and relational (knowing each other) – has a potential to build a bridge across fragmentation. The strength of collaboration between organizations, spanning from loose links or some coordination to full integration [30], likely plays a pivotal role in success.

This study shows that boundary work, which involves informal collaborative efforts with care providers across boundaries, contributes to emergent health service integration and is enhanced by prior familiarity with the providers. These findings are supported by research on team collaboration across borders showing how only teams that manage to span and play with boundaries continue to integrate health services ‘just by doing’ [31]. In various projects included in this meta-synthesis, professionals engaged in informal and experimental ‘boundary play’. This involved showing courage by venturing beyond one’s comfort zones, connecting with professionals on 'the other side' and stretching outside professional and organizational boundaries. The driving force behind such approaches was shared commitment to making things work, for the benefit of patients. Such adaptive work compensates for the one-size-fits-all system that tries to fit a square peg into a round hole, in line with Leutz’s fourth law [27]. However, this is also one of the aspects that complicates the integration of care. For example, digital tools designed to bridge organizational boundaries are rarely integrated with the existing communication systems, leading to increased workloads, as professionals are forced to document everything twice. Further, when using digital communication, professionals need to be sensitive to the situation and each patient’s needs. Best practices are characterized by flexible use of information and communication systems, regardless of their rigidity.

Better integration of care is underlined as a key to tackling the challenges of increasing needs from an aging population, a rising workload for an already overburdened workforce and limited financial resources [8]. Recent evaluations of integrated care initiatives in England [21] and data spanning more than 20 years in the Netherlands [20] make it clear that the challenges of integration persist. These challenges may also be relevant to the Swedish context. According to Nies et al. [20], their study of several healthcare and social care policy reforms indicates that integration is a complex process, demanding greater dynamics than Leutz’s ‘keep it simple, stupid’ principle would imply [27]. In the Dutch context, achieving integration requires continuous efforts to align mechanisms, which may necessitate the consistent application of various policy instruments over a long period of time. Although integrated care holds a promise of improving patient satisfaction and care quality, its overall impact remains multifaceted and context-dependent. In a rapidly changing policy landscape, the clash between centralized governance and the priorities of individual providers may prevent service coordination. The Swedish system features a pragmatic blend of interest representation and policy coordination, enabling each region and municipality to tailor care based on the local context. However, decentralized care systems, such as those in Denmark, Sweden, and soon Finland, face challenges due to substantial local variations, which may jeopardize care equity [26]. In contrast, the corporatist structure and culture of the governance system in the Netherlands make it difficult to identify leaders [20]. These local adjustments align with Leutz’s sixth law: ‘all integration is local’ [27], although they also complicate integration, as best practices in one context are not always suitable in another.

This brings us back to Leutz’s first law, which states: ‘You can integrate some of the services for all the people, all the services for some of the people, but you can’t integrate all the services for all the people’ [27]. This principle may hold the key to making integration easier. Our meta-synthesis also offers insights in this direction, emphasizing flexible care and the importance of a holistic view, and recognizing that not all services are necessary for everyone, at all times. The grounded theory developed by Hedqvist et al. [30] reveals that varying levels of integration are prevalent. Whereas people with extensive needs benefit from comprehensive health service integration, those with occasional urgent care requirements may find coordination or even simple links between services to be sufficient. Unorthodox thinking may be the key to simplifying the integration of services. Recognizing that equity and equality are not the same can challenge standardized care pathways and foster flexible communication and information transfer. It is also vital to remember that trust is built through physical and relational proximity in learning and adaptable organizations. Addressing these complexities and adjusting implementation strategies is not only important, but essential for successful integration efforts.

### Strengths and limitations

To ensure the trustworthiness of the result, an iterative process with expert and research team discussions was employed to ensure that the result was grounded in the included research. Furthermore, for the sake of transparency, the method was described in detail to enable the reader to understand how the results were developed. One of the key strengths of this study was the researcher and data triangulation, i.e., the integration of different kinds of data and perspectives in the synthesis. Conducting the study collaboratively enriched the discussions with multiple perspectives, thereby broadening the understanding of the subject matter. However, there are inherent risks in such an approach, including the need for specific conditions to promote effective cooperation [25]. These conditions were met in the research team, by fostering an open climate and maintaining mutual respect among the researchers and experts.

As the integration of healthcare services is context-dependent [7], one strength of this study is its homogeneous context. We sought to accumulate knowledge on this complex area from multiple individual studies, using different methods and stakeholder perspectives, all conducted in a consistent environment with similar care structures and regulations. By including studies that examine the same problem area, using various qualitative data collection and analysis methods, and involving stakeholders from regional and municipal health and social care services, as well as patients and next of kin, this approach possibly ensures that the accumulation of knowledge is comprehensive and trustworthy within this specific context [24, 32, 33]. A potential limitation could be raised regarding the transferability of the results to other countries. Several contextual aspects need to be considered when transferring results from one context to another, including the practice, the larger organization, and the external environment. To enhance transferability, a description of the context from which these results are derived is therefore provided [34]. Even though healthcare systems differ, there are similarities in the problems faced with integrating care and likely in the solutions as well. The high level of abstraction in the results facilitates their transferability to the conditions of other countries. Moreover, in qualitative research, transferability is described as a collaborative approach between the authors, who present the results, and the reader, who judges how they can be transferred and adapted to their own context [35–37]. A potential aid in judging the transferability of the results is the synthesis of key components, which are not necessarily bound to specific contexts.

However, the CRDA does not claim to encompass all existing evidence. Still, the study’s strength lies in the inclusion of a relatively large number of articles and manuscripts (26 in total), and additional knowledge and experience from collaborating researchers and clinicians as well as the research team members. Another strength is the representation of patients and next of kin participating in about half of the analysed dataset. The remaining data came from various caregiving perspectives, including a coordinator for family carer support, professionals, and managers from various provider organizations, such as regional and municipal primary care and hospital care. This provides a comprehensive description of the components of best practices.

Meta-synthesis is frequently described as a method that produces a 'whole' greater than the sum of its individual parts [38]. The key question to consider is the practical value of this synthesized product: what purpose does it serve? The method used here, however, goes beyond merely aggregating primary studies. It synthesizes findings to build robust evidence that can inform both practice and further research. By integrating and interpreting data from multiple studies, meta-synthesis provides a deeper understanding and generates new insights that individual studies alone may not reveal. This comprehensive approach ensures that the synthesized evidence is not only more holistic but also more applicable and valuable in real-world settings.

## Supplementary Information

Supplementary Material 1.

## Data Availability

All articles included in the analysis are publicly available in the original publications. However, the workshop data are not publicly accessible but can be obtained from the corresponding author upon reasonable request.
