# Supplementary material for: Flexibility in rigid systems: a meta-synthesis of best practices for integrated care
Source: BMC Prim Care. 2025 Nov 11;26:353. doi: 10.1186/s12875-025-03062-y (PMC12604253; doi:10.1186/s12875-025-03062-y)
Supplement: Supplementary file 1 — Supplementary Material 1. [file 12875_2025_3062_MOESM1_ESM.docx]

| Appendix 1, Articles included in the metasynthesis | | | |  |
| --- | --- | --- | --- | --- |
| **Authors, title, scientific journal** | **Aim** | **Design/method/**  **participants** | **Main findings** | **Ethical approval** |
| **1. Adelsjö, I., Nilsson, L., Hellström, A., Ekstedt, M., & Lehnbom, E. C.** (2022). Communication about medication management during patient-physician consultations in primary care: a participant observation study. BMJ open, 12(11), e062148. https://doi.org/10.1136/bmjopen-2022-062148 | To explore communication about medication  management during annual consultations in primary care. | Qualitative descriptive design  *Observations* of primary care consultations between patients (n=18) and general practitioners (n=10).  Data was analysed with qualitative Content analysis. | Four categories became evident, namely: 1) communication barriers 2) striving for a shared understanding of medication management 3) evaluation of the current medication  Treatment 4) the plan ahead and behavioural changes in relation to medication management. Misinterpretations in communication, negligence in informing about changes in medication treatment, and employment of generic alternatives hindered the attainment of a mutual understanding and consensus on continued treatment. | The Regional Ethical Review Board in Linköping  (registration number 2018/109-31). |
| **2. Brandberg, C., Ekstedt, M., & Flink, M.** (2021). Self-management challenges following hospital discharge for patients with multimorbidity: a longitudinal qualitative study of a motivational interviewing intervention. BMJ open, 11(7), e046896. https://doi.org/10.1136/bmjopen-2020-046896 | To describe challenges in self-management activities among people with multimorbidity during a 4-week post-discharge period. | Descriptive and comparative design  Audio-recorded motivational interviewing-sessions between discharge coaches and sixteen participants diagnosed with heart failure or chronic obstructive pulmonary disease and at least one other chronic condition, were included.  Data were analyzed using qualitative content analysis and a longitudinal analysis of changes in self-management process. | Self-management after hospital discharge was a dynamic process with several shifting features that evolved gradually over time. Patients with multimorbidity experienced two major challenges with self-management in the first 4 weeks following hospital discharge: 'Managing a system-centred care' and 'Handling the burden of living with multiple illnesses at home post-discharge'… | The regional Ethical Review Board in Stockholm (registration number 2014/1498-31/2; 2016/735-32) |
| **3. Djukanovic, I., Fagerström, C., Schildmeijer, K., & Tuvesson, H.** (2023). Taking command of continuity-An interview study with agency nurses. Nursing open, 10(4), 2477–2484. https://doi.org/10.1002/nop2.1504 | To describe continuity from the perspective of working as an agency nurse. | Qualitative descriptive design  Individual semi-structured interviews with 15 registered nurses working at Agency companies.  Data were analysed using Ttematic analysis. | Agency nurses are important contributors to continuity through their competence and experience. Further, in a work environment with a high turnover of regular staff, ANs might be the carriers of continuity, ensuring safe and high-quality care. To maintain continuity, ANs considered it vital to take responsibility and be autonomous and self-confident, as this made them step up and lead the work. | The regional Ethical Review Board in Linkoping (registration number 2018/23-31; 2019-04422) |
| **4. Ekstedt, M., & Ödegård, S. (2015).** Exploring gaps in cancer care using a systems safety perspective. Cognition, Technology & Work, 17(1), 5–13. https://doi.org/10.1007/s10111-014-0311-1 | To provide a deeper understanding of how professionals in cancer care prevent medical mishaps by anticipating, discovering and handling gaps that occur in the daily work. | Qualitative descriptive design  Focus group *interviews* with various professionals in cancer care.  Data was analysed using qualitative content analysis. | The study found that ensuring patient safety during cancer care requires an organization that is resilient and capable of anticipating, monitoring, adapting, and learning at all levels of care. Professionals were able to anticipate gaps in situations where there were limited contacts between providers and when faced with time or resource constraints. The ability of professionals to manage these gaps depended on their capacity to adapt to complex and unexpected situations in their daily work. However, managers and clinicians had different perceptions of how to manage these gaps. To improve the continuity of care, patients should participate in decisions about treatments and care plans and take mutual responsibility for transferring information and knowledge across professional boundaries. From a resilience engineering perspective, the results highlight the management's responsibility to address gaps in the system. Designing resilient healthcare organizations allows professionals to prevent human error or reduce its impact. | Not required |
| **5. Ekstedt, M., Schildmeijer, K., Backåberg, S., Ljungholm, L., & Fagerström, C.** (2022). 'We just have to make it work': a qualitative study on assistant nurses' experiences of patient safety performance in home care services using forum play scenarios. BMJ open, 12(5), e057261. https://doi.org/10.1136/bmjopen-2021-057261 | To explore assistant nurses’ adaptive responses to unexpected situations in everyday work to ensure safe care in the home-care context. | Qualitative descriptive design  Drama-based learning and reflection technique *Forum Play* with following group interviews.  The data were analysed using Thematic analysis. | Safety performance in home care service is dependent on the staff who are closest to the older person daily deal with safety risks and ethical dilemmas, having access to information, competence, and resources that fit the demands. | The Swedish Ethical Review Authority  (registration number 2020-01219) |
| **6. Flink, M., & Ekstedt, M.** (2017). Planning for the Discharge, not for Patient Self-Management at Home - An Observational and Interview Study of Hospital Discharge. International journal of integrated care, 17(6), 1. https://doi.org/10.5334/ijic.3003 | To explore how discharge information is prepared and provided to patient in the transition from hospital to home. | Qualitative descriptive design  1)*Observation* of 30 discharge encounters and 2) follow-up individual *interviews* with patients and various healthcare professionals working at hospitals.  Data were analysed using qualitative Content analysis. | Extensive amounts of time, effort, and resources were devoted to preparing the patient's discharge. Both the home-going team and registered nurses meticulously planned the practical and social elements of the patient's departure, while the physicians created a discharge letter using plain language. However, less attention was paid to communicating the actual discharge information to the patients. During discharge encounters, the patients were typically only given an average of 4 minutes and 46 seconds, and the information shared was mainly retrospective in nature, focusing on the hospitalization period, while neglecting self-management tasks and lifestyle advice. | The regional Ethical Review Board in Stockholm (registration number 2014/1498-31/2; 2016/735-32) |
| **7. Freilich, J., Nilsson, G. H., Ekstedt, M., & Flink, M.** (2020). “Standing on common ground” - a qualitative study of self-management support for patients with multimorbidity in primary health care. BMC Family Practice, 21(1). https://doi.org/10.1186/s12875-020-01290-y | The aim of this study was to  explore professionals’, patients’, and family caregivers’ perspectives on how PHC professionals should support selfmanagement  in patients with multimorbidity. | Qualitative descriptive design  Focus group, pair and individual *interviews* with patients, relatives and various healthcare professionals (n=42).  Data was analysed using qualitative Content analysis. | The main theme was “Standing on common ground enables individualized support.” For this support to be successful, professionals had to be cognizant of their personal opinions regarding who is primarily responsible for patients' self-management, as well as the patients' own self-management abilities, needs, and perspectives. The development of personal continuity and trust was beneficial in fostering this understanding. The results also emphasized that experts should be readily accessible to patients with multiple conditions, act as knowledge translators by assisting patients in comprehending their symptoms and their correlation to illnesses, and act as mediators between different levels of care.  The use of telemedicine enabled constant monitoring and facilitated patient interaction with primary healthcare professionals. | The regional Ethical Review Board in Stockholm (registration number 2018/9–31/2). |
| **8. Hedqvist, A. T., Praetorius, G., Ekstedt, M., & Lindberg, C.** (2025). Entangled in complexity: An ethnographic study of organizational adaptability and safe care transitions for patients with complex care needs. Journal of advanced nursing, 81(9), 5528–5545. <https://doi.org/10.1111/jan.16203>  (*published during the manuscripts process)* | To elucidate how organizational adaptability affects patient safety in transitional care for older people with complex care needs | Qualitative descriptive design  Case study. Including document reviews, participant observations and interviews with health and social care personnel.  Data was analysed using Functional Resonance Analysis Method (FRAM). | The findings reveal the importance of timing and precision in coordination of care to maintain patient safety in care transitions. Variability originating from issues of precision is primarily handled by adaptations taking up more resources and time. If there are resources available allowing for flexibility and adjustments, deficient precision can be handled, requiring additional resources that have not been prepared or scheduled. Variability in timing is harder to adjust for since it will affect aspects such as synchronization and access to resources defined by the system design and thus the possibility to achieve quality or precision. Timing issues may cause the system to become more rigid and brittle, making it difficult to adjust to simultaneous variability in precision… | The Swedish Ethical Review Authority  (registration number 2020-01219) |
| **9. Hedqvist, A.-T., Praetorius, G., & Ekstedt, M.** (2023). Exploring interdependencies, vulnerabilities, gaps and bridges in care transitions of patients with complex care needs using the Functional Resonance Analysis Method. BMC Health Services Research, 23(1). https://doi.org/10.1186/s12913-023-09832-7 | The aim of this study was to 1) map coordination and team collaboration across  healthcare and social care organisations, 2) describe interdependencies and system  variability in the discharge process for older people with complex care needs, and to 3)  evaluate the alignment between discharge planning and the requirement at home. | Qualitative descriptive design  Observation, interviews with various healthcare and social care professionals and document review.  The data was analysed using functional resonance analysis method (FRAM). | The discharge process from hospitals is time-sensitive and involves several interdependent factors, and the performance of healthcare professionals is constrained by the system design and organizational boundaries. The most significant vulnerability arises when patients reach their homes because any maladaptation earlier in the care chain can lead to a pile-up of issues for municipal personnel working in health and social care, who are closest to the patients. The personnel's ability to adjust is limited, especially during certain times of the day, which forces them to make compromises to ensure patient safety. However, having appropriate resources and flexibility can help in handling performance variability and responding to uncertainties in care after the discharge | The Swedish Ethical Review Authority  (registration number 2020-01219) |
| **10. Hedqvist, A.-T., Lindberg, C., Hagerman, H., Svensson, A., & Ekstedt, M.** (2024). Negotiating care in organizational borderlands: a grounded theory of inter-organizational collaboration in coordination of care. BMC Health Services Research, 24(1). <https://doi.org/10.1186/s12913-024-11947-4>  (*published during the manuscripts process)* | To construct a grounded theory of how interprofessional collaboration across care providers is performed to support coordination of care for patients with complex care needs | Grounded theory design  *Observations* and *interviews* with various healthcare and social care professionals (n=86).  Data was analysed using constructivist grounded theory. | Interprofessional collaboration in transitional care emerges as a continuum of "Moving from fragmentation to integration through collaborative efforts" spanning from "Dividing care by organizational boundaries", "Establishing paths for collaboration across and within organizational boundaries" to "Co-constructing a comprehensive whole beyond organizational boundaries". The lowest degree of integration relates to the experiences of fragmentation when care is focused on a defined specialist area, obscuring the holistic view. On the next degree, paths for inter- and intraorganizational collaboration is established by coupling, dependent on the interprofessional team's ability to achieve access to each other's knowledge and information. The highest degree of integration is consolidated as the interprofessional team collaborates with the patient and their family as equal parts of the team, by pronounced common goals along with a shared mental map concerning the objectives of care… | The Swedish Ethical Review Authority  (registration number. 2020-01219) |
| **11. Kneck, Å., Flink, M., Frykholm, O., Kirsebom, M., & Ekstedt, M.** (2019). The Information Flow in a Healthcare Organisation with Integrated Units. International journal of integrated care, 19(3), 20. https://doi.org/10.5334/ijic.4192 | To explore information pathways within an integrated healthcare and social care organization and describe how information continuity was delivered for an older patient with complex care needs. | Qualitative descriptive design  A single case study with focus group (n=1) and individual *interviews* (n=6). Various professionals included from hospitals, home care, municipality and primary care.  Data were analysed using qualitative Content analysis. | Partners in care faced hindrances in receiving information, leading to double documentation, alternative information channels, and information being lost. The responsibility of managing and transmitting information between various caregivers rested heavily on patients and their family members. Although patients were expected to play an active role in their care, they were mostly excluded from the flow of information. | The regional Ethical Review Board in Stockholm (registration number 2014/1498-31:2). |
| **12. Lindblad, M., Flink, M., & Ekstedt, M.** (2018). Exploring patient safety in Swedish specialised home healthcare: an interview study with multidisciplinary teams and clinical managers. BMJ Open, 8(12), e024068. https://doi.org/10.1136/bmjopen-2018-024068 | To explore how patient safety in Swedish specialised home healthcare is described and adressed from multidisciplinary teams’ and clinical managers’ perspectives. | Qualitative descriptive design  Focus group *interviews* (N=9) with various professionals in home health care.  Data were analysed using qualitative Content analyse. | The established care ideology that shaped the common mindset of multidisciplinary teams and clinical managers prioritized patient safety. However, the increasing complexity of healthcare posed challenges to this patient safety culture, such as the need to adhere to standardized guidelines, quality assessments, and information management in inadequate communication systems, as well as the demand for required competence and skills. While guidelines and quality assessments from a macro-level aimed to promote patient safety, they limited the ability to adapt to challenges on a meso-level and micro-level. | The regional Ethical Review Board in Stockholm,  (registration number: 2012/1384:31) |
| **13**. Lindblad, M., Unbeck, M., Nilsson, L., Schildmeijer, K., & Ekstedt, M. (2020). Identifying no-harm incidents in home healthcare: a cohort study using trigger tool methodology. BMC health services research, 20(1), 289. https://doi.org/10.1186/s12913-020-05139-z | To explore the cumulative incidence, preventability, types, and potential contributing causes of no-harm incidents that affected adult patients admitted to home healthcare. | Quantitative descriptive design.  A structured retrospective *record review* using a trigger tool designed for home healthcare. A random sample of 600 home healthcare records from ten different organizations across Sweden was reviewed.  Data were analysed using descriptive statistics. | No-harm incidents and potential contributing causes are valuable sources of knowledge for improving patient safety, as they highlight system failures and indicate risks before an adverse event reach the patient. | The Regional Ethics Committee of Linköping (registration number 2014/150–31 and 2016/45–32). |
| **14. Lindblad, M., Flink, M., & Ekstedt, M.** (2017). Safe medication management in specialized home healthcare - an observational study. BMC health services research, 17(1), 598. https://doi.org/10.1186/s12913-017-2556-x | To explore what constitutes the complexity of the medication management process (MMP) in specialized home healthcare and how healthcare professionals handle this complexity. | Grounded theory design  *Observations* and *interviews* with registered nurses in specialized home healthcare (n=27).  The data were analysed using constant comparative methods inspired by the constructivist version of grounded theory. | The MMP in home healthcare was a challenging and intricate system with unclear boundaries of responsibilities, insufficient information systems, and variable working conditions. Healthcare professionals modified their clinical practices by sharing responsibility while maintaining their authoritative roles and respecting patients' active participation, autonomy, and integrity. In order to ensure a safe MMP, healthcare professionals continuously re-priorities goals, handled communication gaps and informational transmission at a distance through novel bridging solutions. Although trade-offs and workarounds were essential, they also jeopardized patient safety as they were not systematically evaluated or devised into learning strategies. | The Regional Ethical Review Committee in Stockholm,  (registration number 2012/1384:31). |
| **15. Ljungholm, L., Klinga, C., Edin-Liljegren, A., & Ekstedt, M.** (2022). What matters in care continuity on the chronic care trajectory for patients and family carers?-A conceptual model. Journal of clinical nursing, 31(9-10), 1327–1338. https://doi.org/10.1111/jocn.15989 | To describe essential aspects of care continuity from the perspectives of persons with complex care needs and their family carers. | Grounded theory design  Individual *interviews* with 16 patients with complex care needs and their relatives.  Data were analysed using Constructivist grounded theory. | A conceptual model of care continuity was developed with five interconnected categories that revolved around the core category: *time and space*. The provision of timely and personalized care was crucial to patients' and family carers' experiences of care continuity where they emphasized the need for access to *tailored information*, irrespective of who was performing a care task, as essential for *mutual understanding*. This necessitated *clarity in responsibilities and roles*, *interprofessional collaboration*, and a *trusting relationship* across the care chain, over *time and space*. All identified categories were essential to achieve care continuity, as they complemented each other, rather than working in isolation. | The regional Ethical Review Board in Linkoping (registration number 2018/23–31). |
| **16. Ljungholm, L., Edin-Liljegren, A., Ekstedt, M., & Klinga, C.** (2022). What is needed for continuity of care and how can we achieve it? – Perceptions among multiprofessionals on the chronic care trajectory. BMC Health Services Research, 22(1). https://doi.org/10.1186/s12913-022-08023-0 | Investigate professionals’ perceptions of the prerequisites of CoC within and between organizations and how CoC can be realized for people with complex needs. | Qualitative descriptive design  Individual paired and focus group *interviews* with various professionals (n=34) in healthcare and social services from three different geographical areas in Sweden.  Data were analysed using qualitative Content analysis. | Professional and cross-disciplinary collaboration at micro, meso, and macro system levels is essential for CoC. Establishing long-term, person-centered relationships at the micro level, maintaining dynamic stability in organizational structures at the meso level, and joint responsibility for cohesive care and enabling uniform solutions for knowledge and information exchange at the macro level are crucial for ensuring continuity. | The regional Ethical Review Board in Linkoping (Dnr 2018/23–31). |
| **17. Schildmeijer, K., Wannheden, C., Nilsson, L., Frykholm, O., Hellström, A., Flink, M., & Ekstedt, M.** (2018). Developing an eHealth Tool to Support Patient Empowerment at Home. Studies in health technology and informatics, 247, 925–929. | To report on the  lessons learned during the initial phases of the development process. | Qualitative descriptive design  Three group *interviews* with a multidisciplinary team of professionals and three semistructured  interviews with prostate cancer patients were performed.  Data were analysed using qualitative content analysis. | Interactive functionality, e.g., reminders and social support, is included to make the ePATH a useful and informative bridge between patients,  next-of-kin and different caregivers. One lesson learned is that it is necessary to  incorporate motivational components in the development of an eHealth tool to  successfully overcome the “intention-behavior” gap. | The regional Ethical Review Board in Linkoping  (registration number 2016/484-31) |
| **18. Schildmeijer, K., Wallerstedt, B., & Ekstedt, M.** (2019). Healthcare Professionals' Perceptions of Risk When Care Is Given in Patients' Homes. Home healthcare now, 37(2), 97–105. https://doi.org/10.1097/NHH.0000000000000717 | To explore health care professionals’ perception of risks in decision making when care is given in older patients’ homes. | Qualitative descriptive design  Focus groups and individual interviews with in total 20 home healthcare professionals were performed.  Data were analysed using qualitative content analysis. | The professionals were aware of taking patient autonomy and involvement in own care into account. Known and unpredictable risks exist in home health care. Priority must be given to creating safer care in this setting. | The Regional Ethical Board of Linköping, Sweden (registration number 2014/150-31). |
| **19. Schildmeijer, K., Frykholm, O., Kneck, Å., & Ekstedt, M.** (2019). Not a Straight Line-Patients' Experiences of Prostate Cancer and Their Journey Through the Healthcare System. Cancer nursing, 42(1), E36–E43. https://doi.org/10.1097/NCC.0000000000000559 | From a patient perspective explore the prostate cancer standardized care pathways, in order to contribute to the understanding of how health care professionals may provide individualized, targeted support at each stage. | Qualitative descriptive design  Individual *interviews* and patient journey mapping were used for data collection.  Data were analysed using qualitative content analysis. | The clinical implementation of the standardized care pathway is  described as a straight path through care, but patients described their experiences as  walking a tightrope. Lack of information, especially about cancer treatment and its  adverse effects, was the most common experience. | The Regional Ethical Board of Linköping, Sweden (registration number 2014/150-31). |
| **20. Skagerström, J., Ericsson, C., Nilsen, P., Ekstedt, M., & Schildmeijer, K.** (2017). Patient involvement for improved patient safety: A qualitative study of nurses' perceptions and experiences. Nursing open, 4(4), 230–239. https://doi.org/10.1002/nop2.89 | To explore nurses’ perceptions and experiences of patient involvement relevant to patient safety. | Qualitative descriptive design  Individual *interviews* with registered nurses (n=11) and nurse assistants (n=8).  Data were analysed using qualitative Content analysis. | Four categories were identified: 1) healthcare professionals’ ways of influencing patient involvement for safer care 2) patients’ ways of influencing patient involvement for safer care 3)barriers to patient involvement for safer care 4) relevance of patient involvement for safer care. The nurses acknowledged that patient involvement is a joint responsibility and stressed that healthcare providers must create opportunities for patients to participate. The nurses also noted that involvement can be impeded by factors related to the patient, healthcare providers, and the healthcare system. Despite this, the respondents emphasized that patient involvement can result in safer care and benefit individual patients. | NA |
| **21. Svensson, I., Von Knorring, M., Hagerman, H., Fagerström, C., Ekstedt, M., & Smeds Alenius, L..** (2023). Unfolding alignment – How top management work to align demand and capacity: an ethnographic study of resilience in a Swedish healthcare region. BMC Health Services Research, 23(1). https://doi.org/10.1186/s12913-023-09291-0 | To explore how top managers work to align demand and capacity in a healthcare region in Sweden. The study is part of a larger project, conducted in a Swedish the region, with the overall aim to study how good leadership is practiced in healthcare. | An ethnographic approach.  *Observations* of management team meetings, individual *interviews* and conversations with top managers.  Data were analysed using qualitative reflexive thematic analysis. | Alignment work was done through active reflection that built on previous experiences and on structures built into the organization at the same time as taking future potential outcomes and consequences into account. In addition to collaborative, preventive, supportive, and contextualizing work, which was conducted in the present, a general approach permeated the organization, which enabled connecting actions, i.e., different forms of alignment work, occurring at different points in time, and connecting different types of knowledge across organizational borders and stakeholders... | The Regional Ethics Review Board in Stockholm  (registration number 2018/1452–31/5). |
| **22. Wibe, T., Ekstedt, M., & Hellesø, R.** (2015). Information practices of health care professionals related to patient discharge from hospital. Informatics for health & social care, 40(3), 198–209. https://doi.org/10.3109/17538157.2013.879150 | To investigate the practices of hospital professionals in providing information to patients and to professionals in primary care at patient discharge from hospital | Qualitative descriptive design  Individual interviews with physicians and registered nurses at hospitals (n=22).  Data were analysed using qualitative Content analysis. | Two themes were identified in the information practices of health care professionals when patients were discharged from hospitals: 1) producing information in parallel processes and 2) challenges in tailoring information to different recipients. | The study was approved by the Data Protection Official at the hospital  and by the Norwegian Social Science Data Services (NSD). As study  participants were HCPs and the study was not concerning personal  health information, an additional approval by the Regional Committee for  Medical and Health Ethics was not needed |
| **23. Strandberg, S., Backåberg, S., Fagerström, C., & Ekstedt, M.** (2023). Self-care management and experiences of using telemonitoring as support when living with hypertension or heart failure: A descriptive qualitative study. International journal of nursing studies advances, 5, 100149. https://doi.org/10.1016/j.ijnsa.2023.100149  (*published during the manuscripts process)* | To describe experiences of self-care management at home when living with chronic illness, with support from primary care through a telemonitoring application | Qualitative descriptive design  Individual *interviews* with 20 patients and 4 informal carers.  The data were analysed using qualitative Content analysis. | The identified theme was: Feeling or not feeling at home with technology as both an intruder and an invited guest. The results of this study revealed that individuals with chronic illnesses who utilized telemonitoring applications at home had the opportunity to enhance their self-awareness and understanding of their bodies. It is recommended to involve informal caregivers in the support team for patients with chronic illnesses, particularly in relation to the utilization of telemonitoring applications at home. Moreover, the findings highlighted that the introduction of telemonitoring applications had an impact on the patient-healthcare professional relationships by offering new channels of communication. | The Swedish Ethical Review Authority (registration number 2019−00889) |
| **24. Ekstedt, M., Nordheim, E. S., Hellström, A., Strandberg, S., & Hagerman, H.** (2023). Patient safety and sense of security when telemonitoring chronic conditions at home: the views of patients and healthcare professionals - a qualitative study. BMC health services research, 23(1), 581. https://doi.org/10.1186/s12913-023-09428-1 | To explore patients’ and healthcare professionals’ experiences of safety and sense of security when using  telemonitoring of chronic conditions at home | Qualitative descriptive design  Individual *interviews* with twenty patients and seven registered nurses and two physicians.  The data were analysed using qualitative Content analysis. | The central theme revolved around experiences of safety and a sense of security was intertwined and relied on patients´ and healthcare professionals´ mutual engagement in telemonitoring and managing symptoms Telemonitoring was seen to enhance symptom awareness and facilitate early detection of deterioration, thereby promoting patient safety. A sense of security was cultivated by having someone keep track of symptoms and encompassed factors such as availability, shared responsibility, technological confidence, and the empowerment of patients in self-management. The meeting with technology changed healthcare professionals’ work processes and patients’ daily routines and brought about potential risks to patient safety, particularly when combined with low levels of health and digital literacy, as well as an uncritical reliance on technology. Empowering patients’ self-management ability and fostering a shared understanding of the patient's health status and symptom control were deemed essential prerequisites for delivering safe care and instilling a sense of security in the patient. | The Swedish Ethical Review Authority (registration number 2019−00889) |
| **25. Ingvarsson, E., Schildmeijer, K., Hagerman, H., & Lindberg, C.** (2024). “Being the main character but not always involved in one’s own care transition” - a qualitative descriptive study of older adults’ experiences of being discharged from in-patient care to home. BMC Health Services Research, 24(1). https://doi.org/10.1186/s12913-024-11039-3 (*published during the manuscripts process)* | To describe older adults’ experiences of being discharged from in-patient care to home. | Qualitative descriptive design  Individual interviews with 17 older adults.  Data was analysed with qualitative content analysis | Even though the older patient is presumed to be the main character in the care transitions they are not experienced as being included in the process. They experience limited understanding and participation in, as well as influence over the care transition. | Swedish Ethical Review Authority (no. 2020–01219). |
| **26. Hagerman, H., Ekstedt, M., Von Knorring, M., Fagerström, C., Tolf, S., & Alenius, L. S.** (2025). Charting the Course Together: Municipal Top-Level Managers: Perspectives on Fostering Safe and Integrated Care for Older Adults Living at Home. International Journal of Integrated Care, 25, 20. https://doi.org/10.5334/ijic.8916    (*published during the manuscripts process)* | To explore municipal top-level managers’ perspectives on fostering safe and integrated care for older adults with complex care needs living at home. | Qualitative descriptive design  Interviews with thirteen top-level managers in municipal social care administration.  Interview data were analysed thematically. | The municipal top-level managers described the importance of establishing individualised, seamless care for older adults living at home without organisational boundaries being evident to the individuals. However, even though the managers were at the top level in their respective organisations, they were also part of a larger system. This required them to collaborate with others, both within and between organisations, in order to meet the increasing demands for care of older adults with complex care needs. | The Swedish Ethical Review Authority (reg. no. 2020-01219) |
